# Von der Viren-Schleuder zum Betreuungsobjekt? Eine Einführung in den Themenschwerpunkt „Kinderrechte in Corona-Zeiten“

**DOI:** 10.1007/s12054-022-00464-5

**Published:** 2022-03-01

**Authors:** Michael Klundt

**Affiliations:** Hochschule Magdeburg-Stendal, Stendal, Deutschland

**Keywords:** Kinderrechte, COVID-19, Corona-Pandemie, Kinderarmut, Kapitalismus, Soziale Polarisierung, Solidarität, Soziale Arbeit

## Abstract

Der einleitende Beitrag skizziert die Entwicklung der Situation von Kindern in den beiden zurückliegenden Jahren der Corona-Pandemie und stellt die Beiträge des Themenschwerpunkts kurz vor.

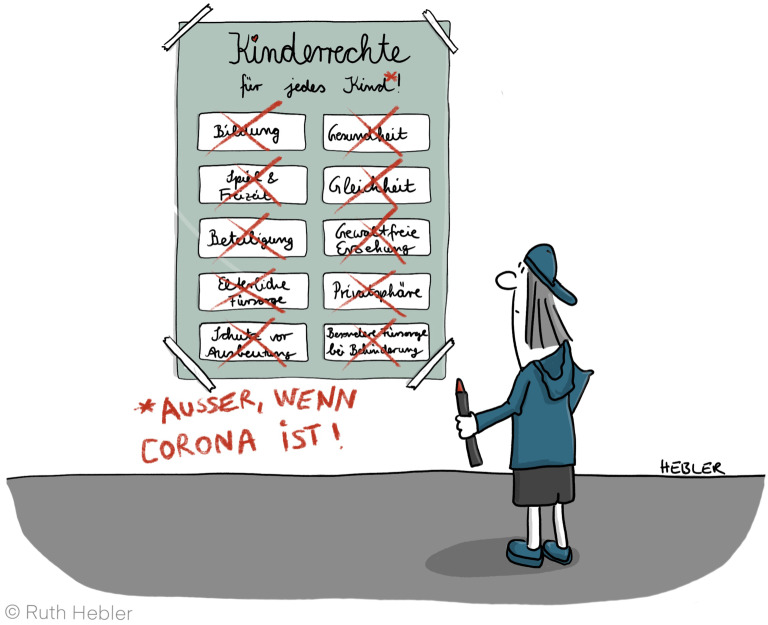



Da die EU-Kommission 2022 zum „Jahr der Jugend“ ausgerufen hat, trifft es sich gut, dass sie intensivere Forschungsanstrengungen über die Folgen der Corona-Pandemie und ihrer jeweiligen Bekämpfung für Kinder und Jugendliche fordert: Das Wissen über die Probleme der jungen Menschen in der Covid-Krise müsse jetzt vertieft werden und es brauche einen evidenzbasierten Ansatz, so die EU-Jugendkommissarin Mariya Gabriel.

Sie sagt: „Besonders problematisch für junge Leute seien nach ersten Analysen die Unterbrechung des Lernprozesses, die Beeinträchtigung psychischer Gesundheit mit Depressionen und Ängsten sowie ein deutlicher Anstieg der Jugendarbeitslosigkeit“ (Evangelisch.de 2022).

Die Weltgesundheitsorganisation WHO und das UN-Kinderhilfswerk UNICEF haben Ende August 2021 noch einmal deutlich hervorgehoben, dass die „katastrophalen Schulschließungen“ der ersten Pandemiewellen seit 2020 zu starken Beeinträchtigungen der seelischen Gesundheit und der sozialen Entwicklung sehr vieler junger Menschen weltweit führten (vgl. UNICEF und WHO 2021). Wie an vielen Beispielen gezeigt werden kann, war und ist das alles nicht (zwangsläufige) Folge von Corona, sondern (auch) dem spezifischen nationalen oder regionalen politischen Krisenmanagement und den jeweiligen sozioökonomischen Kontexten geschuldet (vgl. Klundt 2022, S. 123ff.). Obgleich diverse Auswirkungen global zu beobachten sind, gab und gibt es doch gewisse nationale Unterschiede, die sich auch aus verschiedenen Priorisierungen herleiten lassen.

Was in den beiden zurückliegenden Jahren in meinungsbildenden Medien, Politik und Wissenschaft häufig übersehen wurde, lässt sich inzwischen nicht mehr leugnen. Die Pandemie und die Maßnahmen dagegen (besonders bezogen auf Kinder) sind gleichsam zwei Paar Schuhe. Die Süddeutsche Zeitung vom 29. Dezember 2021 stellte unter dem Titel: „Menschenrechte. Hat Deutschland ein Problem mit Kindern?“ besorgt fest: „Schulschließungen in Rekordlänge, keine Kinderrechte im Grundgesetz, der Skandal um den Kinderpsychiater Winterhoff – da fragt man sich: Welche Rolle spielt das Wohlergehen von Kindern in Deutschland? (…) Testpflicht für Sechsjährige im öffentlichen Nahverkehr während der Schulferien, Sportplatzverbot für Zwölfjährige, die gestern noch elf waren und deshalb noch ungeimpft sind. Verlass ist in der deutschen Pandemiepolitik bisher fast immer darauf gewesen, dass die Kinder in der Debatte um Maßnahmen zunächst mal vergessen wurden.“ Zwar wurden und werden auch in anderen Ländern Kinder funktionalisiert und instrumentalisiert, z. B. hinsichtlich der Beschäftigungsfähigkeit ihrer Eltern (laut SZ-Beitrag am stärksten in Japan, dann in Frankreich und am geringsten in Dänemark, wo Kinder und ihre Rechte bis in die Staatsführung hinein – mindestens verbal – einen offensichtlich relativ hohen Stellenwert besitzen).

Doch die deutschen Rollenzuweisungen für Kinder in den letzten fast zwei Jahren hatten es in sich, so die Journalist_innen der Süddeutschen Zeitung: Erst wurden die Kinder – weitgehend evidenzfrei – als „die“ Viren-Schleudern schlechthin dargestellt und v. a. behandelt. Diese Ausgrenzung von Bildung, Betreuung und Betätigung führte wiederum zur Gefährdung der Beschäftigungsfähigkeit vieler Eltern bzw. konkret: Mütter. Somit erhielten die Kinder, so der SZ-Beitrag, ihre neue Rolle als „Betreuungsobjekt“. Dann wurden Lernschwächen und -verluste immer stärker deutlich, so dass nun Kinder die dritte Funktion zugeschrieben bekamen: die der sog. Leistungserbringer. Doch der Vorrang des Kindeswohls und ihre gesetzlichen Kinderrechte, geschweige denn ihre Mitspracherechte kamen bei alldem deutlich zu kurz. „Geschlossene Schulen, gesperrte Spiel- und Sportplätze, Verbot von Treffen mit Freunden: Die Bilanzen der Verheerung häufen sich. Fast jedes dritte Kind in Deutschland leidet inzwischen an emotionalen Problemen oder Hyperaktivität. Essstörungen, Zwangsstörungen, Angststörungen, depressive Störungen. Die Kinder- und Jugendpsychiatrien des Landes sind noch überlasteter, als sie es vor der Pandemie schon waren.“ (Hahn et al. 2021).

## Wann ist an alle gedacht?

Bemerkbar sind politische Herangehensweisen auch an regierungsamtlichen Werbekampagnen. Im Winter 2020/2021 wurde zum Beispiel in pseudonostalgischen Werbefilmchen der Bundesregierung (aus der Zukunft ins Jahr 2020 wie in Kriegszeiten an die „Front“ zurückblickend) auf fast allen Fernsehsendern im November 2020 unter dem Slogan „besondere Helden“ (offenbar an sog. Kriegshelden anspielend) für das Zuhause-bleiben junger Erwachsener geworben (vgl. https://www.youtube.com/watch?v=krJfMyW87vU). Die jungen Menschen werden dabei aufgefordert, mit Fast Food und Cola vor dem Fernseher regelrecht zu „verschimmeln“, während weder die Sorgen junger Erwachsener um ihren Ausbildungs- oder Arbeitsplatz und ihren Lebensunterhalt, noch die Sorgen derjenigen (meist jungen Leute) berücksichtigt werden, die das Schnell-Essen herstellen, zubereiten und liefern sollen, welches die „besonderen Helden“ vorm Fernseher verzehren.

Ende 2021 war es Zeit für eine neue Werbekampagne. Dass eine erfolgreiche Pandemiebekämpfung und ein gutes Krisenmanagement aus mehr bestehen muss als nur „Impfen“, scheint sich zwar immer mehr herumzusprechen. Dabei ginge es vor allem um politische Anstrengungen zur Verbesserung der Bedingungen im Bildungs‑, Sozial‑, Pflege- und Gesundheitssystem. Nichtsdestotrotz machten das Bundesgesundheitsministerium (BMG), das Robert Koch Institut (RKI) und die Bundeszentrale für gesundheitliche Aufklärung (BZgA) auf riesigen Plakaten, Internet-Homepages und seitengroßen Zeitungs-Annoncen im Dezember 2021 auf mit dem neoliberal konnotierten Slogan: „Wenn alle an ihren Impfschutz denken, ist an alle gedacht“. Doch genau so ist es eben gerade nicht: Es ist leider nicht an alle gedacht worden, wenn alle an ihren Impfschutz denken. Es ist keineswegs ausreichend, von Regierungsseite und deren weisungsverpflichteten Behörden aus zu propagieren, an alle sei dann schon gedacht (vgl. BMG/RKI/BZgA 2021, S. 7). An was z. B. alles nicht gedacht wird, soll im Folgenden gänzlich unvollständig angedeutet werden.

## Verdrängtes Wissen

Als 2019 die Schließung der Hälfte aller Krankenhäuser von einer Bertelsmann-Studie gefordert wurde, sagte der damalige Gesundheitsexperte der SPD, Karl Lauterbach, dazu: „Wir haben schlicht zu viele Krankenhäuser“ (Main Post v. 16.06.^2019^). „Ende Februar (2020) noch hatte Gesundheitsminister Jens Spahn mehr Mut bei Krankenhausschließungen empfohlen.“ (ZEIT v. 07.04.2020>) Dem gelernten Bankkaufmann und Pharma-Lobbyisten war noch vor kurzem Aufrüstung wichtiger als Soziales: „Etwas weniger die Sozialleistungen erhöhen in dem einen oder anderen Jahr – und mal etwas mehr auf Verteidigungsausgaben schauen“, forderte Spahn gegenüber BILD v. 21.02.^2017^. Tafeln und Hartz IV seien sowieso keine Hinweise auf Armut, denn damit habe „jeder das, was er zum Leben braucht“ (Focus.de v. 12.03.2018). Die auch von Spahn und von Lauterbach unterstützten Fallpauschalen und der Marktwettbewerb im Gesundheitssystem bzw. im kapitalistischen Krankheits-Geschäft haben ihren Preis (vgl. Graudenz 2021). Seit 1991 wurden in der BRD über 500 Krankenhäuser geschlossen. Während der Pandemie 2020 machten über 20 Krankenhäuser dicht. Weitere 600 Krankenhäuser sind insolvenzgefährdet. Über 50.000 Beschäftigte fehlen in der Pflege – alles mitverantwortet von der Gesundheitspolitik der Bundesregierung (vgl. junge Welt v. 08.04.^2021^). Deren Konsequenzen lassen sich auch im Feld der Kinder- und Jugendkliniken zeigen: „Seit vor mehr als 25 Jahren die Fallvergütung eingeführt worden ist, mussten bundesweit rund ein Viertel aller Kinderkliniken und Kinderabteilungen aufgegeben werden, 40 % der kinderklinischen Betten wurden abgebaut. Zeitaufwand und Zuwendung, eine Selbstverständlichkeit besonders in der Kindermedizin, kennt das Fallpauschalensystem nicht. Es kennt, wie der Name schon sagt, nur den Fall“, schreibt der ehemalige Chirurg der Klinik in Frankfurt-Höchst, Bernd Hontschik (FR v. 27./28.02.^2021^). Von diesen gesundheitspolitischen Grundlagen las, hörte und sah man in den letzten anderthalb Jahren leider so gut wie nichts in den großen meinungsbildenden Medien.

Dass es die einen oder anderen Probleme bei der Umsetzung der UN-Kinderrechtskonvention vor allem seit Mitte März 2020 gegeben hat, ist inzwischen niemandem mehr verborgen geblieben. Doch wie sah die Gestaltung der Kinderrechte konkret aus? Was bedeutete sie für die Kinderrechte auf Bildung und Gesundheit – vor allem für diejenigen Kinder in Armut(snähe)? Welche Lehren lassen sich für die Vertretung von Kinderrechte-Interessen aus Sicht unterschiedlicher Akteurs-Gruppen ziehen? Welche Entwicklungsprozesse um Kinderschutz und -Beteiligung sind mit der Corona-Krise wie beeinflusst worden? Und schließlich: Was bedeuten diese Entwicklungen für die Soziale Arbeit und die Bestimmungen von Kinderpolitik im Einzelnen wie im Allgemeinen?

Mit diesen und anderen Fragen setzen sich die Autorinnen und Autoren dieses Schwerpunkts auseinander:

- Dabei untersuchen Carolin und Christoph Butterwegge die skandalöse Bildungsungleichheit in Deutschland, ihre Voraussetzungen und gravierenden Folgen für die Betroffenen und die gesamte Gesellschaft.
- Die hessische Kinderrechte-Beauftragte Miriam Zeleke beschreibt die vielfältigen Sichtweisen einiger Heranwachsender auf die Covid-Krise und vergleicht sie mit der Auswertung der JuCo II-Studie für Hessen. Sie zeigt dabei, wie die Rechte von Kindern und Jugendlichen bekannter gemacht und auch in der Praxis einer Pandemie gestärkt werden können.
- Das gleiche Anliegen teilt der Landeskinderbeauftragte Sachsen-Anhalts, Holger Paech, wenn er sich auf Möglichkeiten und Notwendigkeiten einer kinderrechtsbasierten Landespolitik konzentriert. Oberste Regel dabei ist die Befragung der Kinder und Jugendlichen selber, da sie auch die ersten Expert_innen ihres Lebens sind.
- Die beiden Sozialforscherinnen Antje Richter und Gerda Holz untersuchen Kinderrechte, Kinderarmut und Gesundheit in der Corona-Krise.
- Die Sprecherin und der Sprecher des Netzwerks Kinderrechte/National Coalition zur Umsetzung der UN-Kinderrechtskonvention in Deutschland, Bianca Pergande und Jörg Maywald, verdeutlichen den dringenden Bedarf kinderrechtlicher Herangehensweisen in Politik und Gesellschaft.
- Für das Deutsche Kinderhilfswerk (DKHW) formuliert Marie Nadjafi-Bösch Eckpunkte kinderechtsorientierter Kinderpolitik.
- Schließlich überprüft Michael Klundt diverse Dogmen pandemiegemäßer Alternativlosigkeit von Regierungspraxen hinsichtlich der COVID-Krise und der Kinderrechte.

In den Worten des Berichts der interministeriellen Arbeitsgruppe aus Bundesgesundheits- und -familienministerium (IMA) vom 15. September 2021 stellen sich die Folgen der Pandemie und der Maßnahmen folgendermaßen dar: „Die Pandemie hat bestehende Ungleichheiten hinsichtlich der Chancen auf ein gesundes Aufwachsen verschärft. Diejenigen Kinder und Jugendlichen, die bereits vor der Pandemie erhöhte Gesundheits- und Entwicklungsrisiken getragen haben, waren und sind auch während der Pandemie besonderen Belastungen ausgesetzt. Neben Kindern und Jugendlichen aus Familien mit niedrigem sozioökonomischem Status gehören dazu insbesondere auch Kinder und Jugendliche mit Behinderungen und schweren chronischen Erkrankungen mit komplexem Unterstützungsbedarf sowie Kinder und Jugendliche, deren Eltern an schweren psychischen Erkrankungen leiden.“ (BMFSFJ/BMG 2021, S. 19f.) Für diese vulnerablen Gruppen habe die Aufrechterhaltung des Regelbetriebs von Bildungs- und Betreuungseinrichtungen – auch bei einer Dynamisierung des Infektionsgeschehens – eine besondere Bedeutung und Dringlichkeit, so der Bericht. „Sie sind für ihre gesunde Entwicklung in erhöhtem Maße auf verlässliche außerfamiliäre Strukturen angewiesen und können die Belastungen durch den Wegfall dieser Strukturen häufig schlechter kompensieren. Zudem hat sich gezeigt, dass Notbetreuungsangebote von vulnerablen Gruppen, auch aus Angst vor Stigmatisierung, wenig genutzt wurden. Dies soll entsprechend an die zuständigen Länder und Kommunen adressiert werden.“ (BMFSFJ/BMG 2021, S. 20).

Wenn jedoch ab 2023 die „schwarze Null“ und die Schuldenbremse wieder eingehalten werden sollen, stellt sich nicht nur zur Umsetzung von Kinderrechten und zur Bekämpfung von Kinderarmut die Frage, wie notwendige Investitionen in Brücken, Bildung und Bürgergeld adäquat finanziert werden sollen. Die nächste Spar- bzw. Kürzungsrunde scheint dagegen dann bereits vor der Tür zu stehen. Sofern keine Vermögensteuer eingeführt, keine Bürgerversicherung die Zwei-Klassenversorgung im Gesundheits- und Pflegesystem beenden, wenn mit der gesetzlichen Rente an der Börse spekuliert werden und der Rüstungsetat derweil steigen soll, steht der gewagte Fortschritt der Ampelkoalition auf ziemlich wackeligen Beinen. Die Soziale Arbeit wird sich in diese Kräfteverhältnisse mit ihrem politischen Mandat einmischen müssen – und sei es auch nur, um die nächsten Tarifrunden für die Sicherung und Verbesserung der Bedingungen in den Sozial- und Erziehungsberufen solidarisch zu gestalten.
